# Mental Health Literacy and Education of Complementary Medicine Practitioners: A Cross-Sectional Study

**DOI:** 10.1007/s10488-023-01339-x

**Published:** 2024-01-18

**Authors:** Joanna E. Harnett, Matthew J. Leach, Randa Karzon, Erica McIntyre

**Affiliations:** 1 Faculty of Medicine and Health School of Pharmacy, The University of Sydney, Building A15, Science Rd, Camperdown, NSW 2006 Australia; 2 National Centre for Naturopathic Medicine, Southern Cross University, Rifle Range Rd, Lismore, NSW 2054 Australia; 3 Endeavour College of Natural Health, Fortitude Valley, QLD 4006 Australia; 4 Institute for Sustainable Futures, University of Technology Sydney, Sydney, NSW Australia; 5 Research Institute for Innovative Solutions for Wellbeing and Health, Faculty of Health, University of Technology Sydney, Box 123, Broadway, NSW 2007 Australia

**Keywords:** Mental health services, Practitioner mental health literacy, Practitioner education, Complementary medicine

## Abstract

An estimated 42% of Australians who consult complementary medicine (CM) practitioners have a mental health diagnosis. Preparedness of CM practitioners in managing such diagnoses is currently unknown. A cross-sectional survey of 257 CM practitioners who reported caring for people with a mental health diagnosis. Practitioners’ mental health literacy, educational needs, and confidence in the assessment, management, and treatment of mental health—including suicide risk—were analysed. Most (59.1%) participants had no formal qualifications in mental health and 44.3% indicated they had not completed any training in psychological therapies. Only 20% were trained in mindfulness-based techniques or goal setting. Over 50% reported their undergraduate qualification contained insufficient mental health content to prepare them for clinical practice. Over one-half had attended continuing professional education on mental health. Practitioners reported greater confidence in assessing, managing, and treating mental wellbeing over complex mental health disorders and suicide risk. These findings uncovered a deficit in the CM practitioner’s surveyed mental health education. As these CM practitioners are a primary point of contact for patients with mental health diagnoses, there is a critical need to expedite skills development in this workforce to support the delivery of safe and effective primary mental health care.

**Supplementary Information:**

The online version contains supplementary material available at 10.1007/s10488-023-01339-x.

## Introduction

Australian adults are high users of complementary medicine (CM), with over one-third (36%) consulting a CM practitioner within a 12-month period. Of these, 10% consulted a naturopath or Western herbalist (Steel et al., 2018). Over 42% of those visiting these CM practitioners reported having a mental health diagnosis (McIntyre et al., 2021). Collectively, the out-of-pocket spending on CM services, products and practices by those with a mental health diagnosis was estimated to be AU$1.18 billion (US$880 million) (McIntyre et al., 2021). The high prevalence of CM use among people with chronic and mental health disorders is claimed to be partly driven by unmet healthcare needs (McIntyre et al., 2021; Steel et al., 2018).

CM practitioners (specifically naturopaths, Western herbalists, and nutritionists [non-dietetic]) are trained to use a range of therapies to support the prevention and management of diverse chronic conditions, including mental health disorders (Steel, Schloss, Steel et al., 2020a, b). These therapies may include dietary advice, herbal medicines, homoeopathic and nutritional supplements, lifestyle education and behaviour change, mind body therapies (e.g., visualisation, meditation, breathing techniques), and counselling (Steel, Foley, Steel et al., 2020a, b; Steel, Schloss, Steel et al., 2020a, b). However, the utilisation of these practices varies within and across these three CM disciplines, as do the education standards for these professions (Dunn et al., 2021; Wardle et al., 2012).

Naturopaths, Western herbalists, and nutritionists (non-dietetic) are not statutory regulated and thus, are not governed by the Australian Health Practitioner Regulation Agency (AHPRA). Instead, these professions are self-regulated by professional associations, which provide accreditation and governance of the profession and the practitioners they represent (Dunn et al., 2021). Each professional association has a code of conduct to define a practitioner’s scope of practice, and the need to recognise and refer a person with conditions that are outside a practitioner’s expertise to manage and treat. As outlined in a ‘2022 Health Technology Assessment – Naturopathy’, naturopaths across the globe have a broad scope of practice and ‘*employ a range of assessment tools including thorough case history taking, standard conventional physical examinations and laboratory testing’*; and that the scope of practice includes among other things ‘*identifying factors contributing to a patient’s state of health, their symptoms and/or diseases.’*(Lloyd I., 2021).

While there have been some attempts to understand the types of therapies that this sub-group of CM practitioners prescribe, and the conditions they treat, there has been limited investigation into their preparedness to manage such conditions (particularly mental health disorders). Examining practitioner preparedness for practice is of importance to public health as it can establish whether practitioners pose any risk of harm to people living with mental health disorders, which in turn, can help identify opportunities to improve patient outcomes and experiences (McIntyre, 2016). For instance, practitioners with inadequate education and training may represent a significant public health concern as inappropriate treatment, and/or delayed or inaccurate diagnosis, can increase the risk of developing a more serious mental illness (Jorm, 2012). Similarly, an increase in practitioner mental health literacy has been shown to have a positive impact on patient outcomes through improvements in appropriate referral behaviour and a reduction in inappropriate patient referrals (i.e., referrals to emergency services)(McCaffrey et al., 2017).

Determining the mental health literacy and education of non-AHPRA regulated CM practitioners who are likely to recommend ingestible therapies is a critical first step to understanding the preparedness of this workforce to care for people living with mental health disorders. By identifying mental health knowledge/skills deficits in this important sub-group of CM practitioners, and implementing appropriate strategies to address these deficits (e.g., practitioner training, changes to education standards), These practitioners may be able to make an effective and safe contribution to this important area of primary healthcare (Dunn et al., 2021). This is particularly important given the increased need for mental health services, and current health workforce shortages in Australia.

## Methods

### Design

The study was a national cross-sectional survey. The study was reported in accordance with the Strengthening the Reporting of Observational Studies in Epidemiology (STROBE) Statement.

### Aims and Objectives

The study aimed to examine the mental health literacy (concept 1) and decision-making and practice behaviours (concept 2) of Australian CM practitioners to determine their preparedness to manage common mental health problems and disorders; specifically, CM practitioners who are not statutory regulated and are likely to recommend ingestible therapies, including naturopaths, Western herbalists, and nutritionists (non-dietetic).

The specific objectives were to:

- determine the qualifications and training in mental health and psychological therapies among a sub-group of non-statutory regulated CM practitioners;
- ascertain the resources that a sub-group of non-statutory regulated CM practitioners employ to inform mental health clinical decision making; and.
- assess the mental health literacy i.e., perceived knowledge and confidence to provide mental health care, of a sub-group of non-statutory regulated CM practitioners who clinically manage common mental health problems and diagnosed disorders.

### Recruitment and Data Collection

Non-AHPRA regulated CM practitioners (i.e., naturopaths, Western herbalists, and nutritionists [not accredited practicing dietitians]), who were financial members of any CM professional association, practiced in any Australian state or territory, and consulted patients for mental health related conditions, were eligible to participate in the survey. Non-probability convenience sampling was used to recruit this sub-group of CM practitioners via the practice-based research network (PRACI) database, CM professional associations, and social media (e.g., Twitter, Facebook) between July and December 2019. After clicking on a link to the online survey, respondents were presented with information about the research (e.g., purpose, benefits and risks), and ethical considerations (e.g., confidentiality, anonymity, right to withdraw). Completion of the survey was indicative of implied informed consent. Based on an estimated target population of 5,000 CM practitioners (i.e., naturopaths, Western herbalists, and nutritionists) (Leach & Bugarcic, 2021; Leach et al., 2014), a 7% margin of error and 95% confidence for any individual item on the survey, we aimed to survey at least 189 participants.

### Survey Instrument

The survey was hosted and stored within the online platform Qualtrics. The 42 survey items solicited information on the mental health care provided in clinical practice, including patient care seeking behaviour i.e., type of mental health problem or disorder, and perceived expectations for mental health care, as well as practitioner characteristics, including qualifications and training in mental health, mental health literacy i.e., perceived level of knowledge and clinical skills related to the treatment and prevention of mental health, and decision-making and practice behaviours associated with managing mental health, and practitioner demographics.

Sociodemographic questions were based on previous items used in surveys of health care professionals and including qualifications held, primary clinical discipline, practice context, and years of practice. Items measuring concept one (mental health literacy) were informed by previous research assessing mental health literacy in health professionals (O’Connor & Casey, 2015; Sivakumar et al., 2011). Items for the second concept (decision-making and practice behaviour) were informed by previous research on CM practitioners (Harnett et al., 2022; Morgan & Francis, 2008), and guided by the content expertise of the research team i.e. practitioners, educators, and researchers in CM and mental health. The survey was pilot tested in a sample of 10 CM practitioners known to the research team to identify any issues related to clarity, face validity, and survey utility, and to confirm the estimated survey completion time. Edits were made as recommended, that primarily related to how participants comprehended the items. The survey took approximately 20 min to complete.

### Ethical Statement

The study was approved by the University of Technology Human Research Ethics Committee (HREC REF NO. ETH19-3477) and was carried out in accordance with the *National Statement on Ethical Conduct in Human Research *(2007).

### Data Analysis

Data were analysed using IBM SPSS Statistics for Windows (v.25.0, IBM Corp., Armonk, N.Y., USA). Multiple responses from single participants were handled using the de-duplication procedure for online surveys (Konstan et al. 2005). Missing data were reported as missing. Responses with > 90% unanswered items were excluded from analyses. Chi square tests were used to analyse associations between nominal-level variables, with Cramer’s V used to determine the strength of associations. Associations between ordinal-level variables were tested using Kendall’s tau correlation coefficient. We interpreted coefficients as follows: 0.10–0.29 (weak association), 0.30–0.49 (moderate association), and 0.50-1.00 (strong association) (Cohen 1988). The level of significance was set at *p* < .05.

## Results

A total of 301 participants initiated the survey. Sixty-four responses were > 95% incomplete and were omitted from the analysis, resulting in an analysable sample of 237 participants. As the reach of the survey could not be quantified, it was not possible to generate a survey response rate.

### Characteristics of Participants

Participants had a mean age of 46.3 (*SD* = 10.98) years and were predominantly female (70.9%; Table 1). Most (60.3%) practiced naturopathy as their primary clinical discipline. The largest proportion of participants worked in solo practice (39.7%), or a practice with other CM providers (30.4%), primarily in the states of New South Wales (22.8%), Victoria (21.1%) or Queensland (19.8%). Practitioners held a range of qualifications, with more than one-half holding a Diploma/Advanced Diploma (57.0%) and/or a Bachelor degree (54.4%). In terms of CM qualifications, the vast majority of participants completed their undergraduate CM qualification through a private college (81.0%), with 45.1% completing their highest CM qualification within the past 10 years. Almost one-half (47.2%) of participants reported holding a qualification in another health discipline, with 24.5% being a qualification in another field of CM.

**Table 1:** Frequencies and percentages for characteristics of participants (*N* = 237)

Participant characteristic	*n* (%)
**Gender**	
Female	168 (70.9)
Male	26 (11.0)
Other	3 (1.3)
Missing	40 (16.9)
**State/territory of practice location**	
New South Wales	54 (22.8)
Victoria	50 (21.1)
Queensland	47 (19.8)
Western Australia	30 (12.7)
South Australia	8 (3.4)
Australian Capital Territory	5 (2.1)
Tasmania	4 (1.7)
Northern Territory	1 (0.4)
**Primary clinical discipline**	
Naturopathy	143 (60.3)
Nutrition	29 (12.2)
Other	19 (8.0)
Western herbal medicine	8 (3.4)
Missing	38 (16.0)
**Type of clinical practice setting**	
Solo practice	94 (39.7)
Practice with other CM providers	72 (30.4)
Integrative practice with a mix of CM and conventional healthcare providers	31 (13.1)
Practice with other conventional healthcare providers	15 (6.3)
Within an educational setting	13 (5.5)
Other	7 (3.0)
Missing	5 (2.1)
All qualifications held*, n (%)	
Certificate IV	24 (10.1)
Diploma/Advanced Diploma	135 (57.0)
Bachelor degree	129 (54.4)
Graduate Certificate/Diploma	49 (20.7)
Master’s degree	31 (13.1)
PhD/Doctorate	10 (4.2)
**Institution of undergraduate CM qualification**	
Private College	192 (81.0)
University	45 (19.0)
**Years since receiving highest CM qualification**	
Less than 5 years	56 (23.6)
5–9 years	51 (21.5)
10–14 years	43 (18.1)
15–19 years	24 (10.1)
20 years or more	24 (10.1)
Missing	39 (16.5)
**Other health qualifications**	
Do not have another health qualification	91 (38.4)
Other CM	58 (24.5)
Allied health	37 (15.6)
Nursing	11 (4.6)
Medicine	6 (2.5)
Missing	34 (14.4)

Note. *Multiple-response item; CM = Complementary medicine

The vast majority of participants had provided some form of care to patients to address their mental health needs, with 76.8% doing so always or very often, and 22.4% doing so sometimes or rarely.

### Concept 1: Mental Health Literacy - Qualifications and Training

The majority (59.1%) of participants reported no formal qualifications in mental health (Table 2). Similarly, 44.3% of participants indicated they had completed no training in psychological therapies, with only 1 in 5 participants undertaking training in mindfulness-based techniques (22.4%), or goal setting (20.3%). In contrast, the majority of participants had undertaken some level of training in mental health, with industry-led (52.7%) or professional association-led (38.4%) seminars/workshops on mental health being the most frequently reported. Completion of formal qualifications in mental health was found to be weakly negatively associated with number of years since receiving the highest qualification in CM (Cramer’s V = − 0.285, *p* = .006). There was also a weak positive association between completion of formal qualifications in mental health and primary clinical discipline (Cramer’s V = 0.238, *p* = .022), with a significantly greater proportion of participants with a primary discipline in naturopathy completing such training.

**Table 2:** Percentages and frequencies for participant qualifications and training in mental health and psychological therapies (*N* = 237)

Type of mental health training or qualification	Participants *n (%)*
**Formal qualifications in mental health**	
Have no formal qualification in mental health	140 (59.1)
Other formal qualifications in mental health	35 (14.8)
Diploma of counselling	13 (5.5)
Bachelor of psychology (3-year)	7 (3.0)
Bachelor of mental health or equivalent	7 (3.0)
Graduate Diploma in mental health / counselling	7 (3.0)
Graduate Certificate in mental health / counselling	6 (2.5)
Advanced diploma of counselling	5 (2.1)
Bachelor of psychology (4-year)	5 (2.1)
Master of psychology (health / clinical / neuropsychology / counselling)	5 (2.1)
Bachelor of counselling	4 (1.7)
PhD in psychology	1 (0.4)
**Training in psychological therapies**	
Have not completed any psychological therapy training	105 (44.3)
Mindfulness-based techniques	53 (22.4)
Goal setting	48 (20.3)
Motivational interviewing	38 (16.0)
Cognitive behavioural therapy	30 (12.7)
Behavioural activation	11 (4.6)
Other psychological therapy training	10 (4.2)
**Training in mental health**	
Industry-led seminar/workshop on mental health	125 (52.7)
Professional association led seminar/ workshop on mental health	91 (38.4)
Academic conference workshop	72 (30.4)
Mental health first aid	58 (24.5)
Professional association led conference workshop	46 (19.4)
Other mental health training	38 (16.0)
Have not completed any mental health training	28 (11.8)

### Concept 1: Mental Health Literacy S- self-efficacy

One-half (50.3%) of participants strongly/somewhat disagreed (27.9% strongly/somewhat agreed) that their undergraduate qualification contained sufficient content on mental health. Similarly, 50.8% participants strongly/somewhat disagreed (29.5% strongly/somewhat agreed) that their undergraduate qualification adequately prepared them to manage mental health problems/disorders in clinical practice. Correspondingly, 64.5% of participants strongly/somewhat agreed (4.7% strongly/somewhat disagreed) that they would have preferred to have undertaken more training in mental health during their undergraduate education. Most (57.8%) participants also strongly/somewhat agreed (and 10.6% strongly/somewhat disagreed) that they would like to undertake postgraduate training in mental health. There were no statistically significant associations between participant perceptions of their undergraduate qualification (i.e., level of content on, or preparedness for managing mental health problems/disorders in practice) and number of patients cared for per week for mental health problems/disorders.

Participants reported to be somewhat/highly confident in their knowledge/skills to manage most mental health problems or disorders, particularly general mental wellbeing (76.4%), anxiety symptoms (74.2%) and sleep problems (70.5%; Table 3). However, for bipolar related conditions and psychotic disorders, a large proportion of participants reported to be either not at all confident or only slightly confident in their knowledge/skills (49.4% and 61.2%, respectively).

**Table 3:** Participant confidence in their knowledge and skills to assess/treat/manage mental health problems or disorders (*N* = 237)

Mental health problem or disorder	Not at all confident	Slightly confident	Moderately confident	Somewhat confident	Highly confident	Missing
	n (%)					
General mental wellbeing	2 (0.8)	3 (1.3)	12 (5.1)	72 (30.4)	109 (46.0)	39 (16.5)
Anxiety symptoms	1 (0.4)	4 (1.7)	17 (7.2)	83 (35.0)	93 (39.2)	39 (16.5)
Sleep problems	1 (0.4)	4 (1.7)	26 (11.0)	85 (35.9)	82 (34.6)	39 (16.5)
Psychological stress-related symptoms	5 (2.1)	13 (5.5)	30 (12.7)	78 (32.9)	71 (30.0)	40 (16.9)
Insomnia disorders	4 (1.7)	17 (7.2)	39 (16.5)	82 (34.6)	55 (23.2)	40 (16.9)
Depression symptoms	0 (0.0)	12 (5.1)	52 (21.9)	77 (32.5)	55 (23.2)	41 (17.3)
Anxiety disorders	5 (2.1)	17 (7.2)	44 (18.6)	78 (32.9)	53 (22.4)	40 (16.9)
Depression disorders	9 (3.8)	39 (16.5)	55 (23.2)	63 (26.6)	30 (12.7)	41 (17.3)
Assessing a patient for suicide risk	25 (10.5)	28 (11.8)	54 (22.8)	63 (26.6)	28 (11.8)	39 (16.5)
Eating disorders	44 (18.6)	46 (19.4)	60 (25.3)	36 (15.2)	12 (5.1)	39 (16.5)
Bipolar related disorders	67 (28.3)	50 (21.1)	45 (19.0)	27 (11.4)	9 (3.8)	39 (16.5)
Psychotic disorders	99 (41.8)	46 (19.4)	39 (16.5)	8 (3.4)	5 (2.1)	40 (16.9)

In addition to assessing self-reported knowledge of suicide risk (of which 49.4% of participants reported to be moderately/somewhat confident) [Table 3], participants were asked to rate the level of suicide risk for different mental health disorders. Most participants believed there was a moderate-high to high risk of suicide for major depression (78.1%), psychotic disorders (72.1%), post-traumatic stress disorder (66.2%), bipolar disorder (61.2%) and substance abuse disorders (63.8%) (Table 4). One-half (50.7%) of participants believed impulse control disorders had a moderate to moderate-high suicide risk, while generalised anxiety disorder was perceived by most participants as having a low-moderate to moderate risk (56.5%).

**Table 4:** Perceived risk of suicide for different mental health disorders (*N* = 237)

Mental health disorder	Low risk	Low-moderate risk	Moderate risk	Moderate-high risk	High risk	Missing
	*n* (%)					
Major depression	0 (0.0)	1 (0.4)	9 (3.8)	49 (20.7)	136 (57.4)	35 (14.8)
Psychotic disorders	1 (0.4)	6 (2.5)	17 (7.2)	69 (29.1)	102 (43.0)	35 (14.8)
Post-traumatic stress disorder	0 (0.0)	5 (2.1)	34 (14.3)	65 (27.4)	92 (38.8)	35 (14.8)
Bipolar disorder	2 (0.8)	11 (4.6)	37 (15.6)	72 (30.4)	73 (30.8)	35 (14.8)
Substance abuse disorders	1 (0.4)	6 (2.5)	38 (16.0)	80 (33.8)	71 (30.0)	35 (14.8)
Impulse control disorders	4 (1.7)	26 (11.0)	67 (28.3)	53 (22.4)	44 (18.6)	35 (14.8)
Generalised anxiety disorder	4 (1.7)	55 (23.2)	79 (33.3)	43 (18.1)	13 (5.5)	35 (14.8)

### Concept 2: Decision-Making and Practice Behaviours - Clinical Decision-Making Resources

Participants used a range of resources to assist their clinical decision-making when managing patients with mental health problems/disorders. The only resource used by more than one-half (52.2%) of participants was peer-reviewed journal articles. Other resources used by participants included textbooks (46.5%), technical information from industry (37.5%), the *DSM* (21.6%), clinical guidelines (19.6%), other (10.6%), and the *ICD* (4.7%).

## Discussion

This is the first known study to investigate the preparedness of a sub-group of non-APHRA registered Australian CM practitioners, predominantly naturopaths, in providing mental health care. The majority of these practitioners reported receiving insufficient mental health education during their undergraduate CM training, and most had not completed any formal qualifications in mental health. While most of the participants reported to be confident in assessing, managing and treating mental wellbeing, common mental health symptoms and less serious disorders, most demonstrated knowledge/skills deficits in information acquisition and aspects of mental health literacy (e.g. determining risk of suicide), as well as low self-efficacy in managing serious mental health disorders.

While the CM context of this study is novel, studies assessing mental health knowledge and literacy in other healthcare practitioners (e.g. nurses, physicians, surgeons, psychologists) have revealed similar findings (Al-Yateem et al., 2018; Worsfold & Sheffield, 2018; Wu et al., 2017). This suggests that these knowledge/skills gaps are not necessarily limited to CM practitioners. Accordingly, examining mental health knowledge and literacy across the broader non-mental healthcare practitioner workforce is warranted to inform educational standards across a range of contexts and countries. This view aligns with the WHO Mental Health Gap Action program, which has identified the presence of ‘*a worldwide gap between service need and provision*’ (Keynejad et al., 2018), and subsequently, has recommended assessment of the mental health literacy and education needs of the primary healthcare workforce.

Many Australians with a mental health diagnosis seek the care of a CM practitioner (McIntyre et al., 2021). It is therefore of concern that most of the practitioners in this study held no formal undergraduate or postgraduate qualification in mental health, or training in psychological therapies. Indeed, inadequate provision of formal training in mental health places both CM practitioners and the people seeking their care in a vulnerable position (Kohn et al., 2022). The CM industry and CM professions including those representing naturopaths, Western herbalists, and nutritionists have attempted to offer continuing professional education (CPE) training opportunities to support practitioner knowledge and skills development related to the management of mental health conditions. However, as most participants in this study did not regularly refer to standard mental health care resources for the management of mental health conditions, this raises questions as to whether these CPE activities enable this sub-group of CM practitioners to develop the necessary knowledge and skills to deliver safe and appropriate mental health care. The perceived inadequacy of current CPE activities is supported to some extent by a recent education needs analysis of Australian naturopaths, which identified mental health as one of the most valued and important areas for future knowledge and skills development (Leach & Bugarcic, 2021).

The dearth of mental health content in undergraduate medical and nursing curricular has been an issue of concern for some time (Acharya et al., 2016; Happell et al., 2015). However, only now has this been identified as a matter of concern for undergraduate CM education including naturopaths. It is possible that this matter may have gone unchecked for so long as the majority of educational institutions providing undergraduate CM qualifications across the globe do not evaluate their educational processes, nor the effectiveness or outcomes of the training they provide (Gray et al., 2019). In addition, standards for naturopathic education (at least in Australia) have been somewhat limited, and have lacked sufficient detail in order to meaningfully shape curriculum (Leach, 2021). Fortunately, recent accreditation standards for naturopathic courses in Australia now specify the need for such curricular to include subject matter on counselling, psychological science, and psychological diagnostic and clinical skills (Australian Register of Naturopaths and Herbalists, 2021) While the impact of these changes remains to be seen, it does represent a positive move forward in preparing CM practitioners such as those represented in this study to care for people with a mental health diagnosis.

Despite current gaps in mental health education, most participants in this study reported to be confident in their knowledge and skills to assess, manage and treat common aspects of mental health, including general mental wellbeing, symptoms of anxiety and sleep issues. Practitioner confidence in the clinical management of more complex or serious mental health disorders, including psychotic illnesses and bipolar disorder, was considerably lower. These self-reported confidence ratings indicate that most practitioners in this study were aware of their scope of practice, as well as the limitations of their knowledge and skills to effectively and safely manage serious and complex mental health disorders. This finding is consistent with previous studies, which have shown the scope of practice of Australian naturopaths with respect to mental health care to be limited to less serious mental health conditions (Steel et al., 2020). Notwithstanding, it is possible that adequate undergraduate or postgraduate mental health training may help improve the self-efficacy of CM practitioners to manage more serious mental health conditions, which could provide a possible solution to current mental health workforce shortages in Australia and globally.

This study is not without limitations. First, the use of convenience sampling raises the risk of self-selection bias, meaning that the sample possibly may not be fully representative of all three of the CM practitioner groups of interest, noting that there was a relatively high proportion of naturopathic practitioners informing the findings of this study. Notwithstanding, the gender and geographical distribution of participants, and the proportion of participants that identified as a naturopath and held a postgraduate qualification, closely approximated that of the Australian naturopathic and Western herbalist workforce (Leach et al., 2014). Second, given that one-half of participants had completed their highest CM qualification ten or more years ago, it is possible that participants may not have accurately remembered the extent to which mental health was included in their undergraduate education, suggesting that participant responses may be impacted by recall bias. In addition, given the survey instrument was developed with two main concepts/themes in mind, health literacy and decision making, and practice behaviours, the individual survey items cannot be interpreted as measures of discrete competencies.

## Conclusions

Although a high proportion of people consult CM practitioners for mental health care, the practitioners included in this study were predominantly naturopaths and they indicated their undergraduate education inadequately prepared them for the provision of such care. Thus, in the interests of public health, there is a need to address deficits in the mental health literacy of naturopaths, as well as improve practitioner confidence in the assessment, management, and treatment of common mental health disorders. Addressing these knowledge/skills deficits will help mitigate the risk of harm to patients, foster improvements in patient outcomes, and support the role of naturopaths in managing the unmet healthcare needs of persons living with mental health disorders.

### Electronic Supplementary Material

Below is the link to the electronic supplementary material.

Supplementary Material 1
