# Supplementary material for: Mental Health Literacy and Education of Complementary Medicine Practitioners: A Cross-Sectional Study
Source: Adm Policy Ment Health. 2024 Jan 18;51(2):217–25. doi: 10.1007/s10488-023-01339-x (PMC10850010; doi:10.1007/s10488-023-01339-x)
Supplement: Supplementary file 1 — Supplementary Material 1 [file 10488_2023_1339_MOESM1_ESM.docx]

The clinical management of mental health by Australian complementary medicine practitioners

Survey Flow

Standard: Informed Consent (1 Question)

Standard: Screening questions (3 Questions)

Standard: Definition of terms used in this study (1 Question)

Standard: Care seeking for mental health (6 Questions)

Standard: Section 2: Clinical management of mental health (5 Questions)

Standard: Section 2b: Clinical management of mental health: treatments used (5 Questions)

Standard: Section 2c: Clinical management of mental health: treatment decision-making (7 Questions)

Standard: Section 3: Interprofessional communication (5 Questions)

Standard: Section 4a: Qualifications and training in mental health (5 Questions)

Standard: Block 9 (4 Questions)

Standard: Section 5: Demographics and practice characteristics (11 Questions)

Standard: End (1 Question)

| Page Break |  |
| --- | --- |

Start of Block: Informed Consent

IP
**ETH19-3477** **The clinical management of mental health by Australian complementary medicine practitioners**
 **What is the research study about?**
 The purpose of this research/online survey is to explore how Australian naturopaths, Western herbalists and nutritionists (not Accredited Practicing Dietitian) approach the clinical management of people living with mental health problems.

 **Who is conducting this research?**
 My name is Dr Erica McIntyre and I am an academic at UTS. I am collaborating on this research with academics Dr Joanna Harnett (The University of Sydney), Dr Matthew Leach (University of South Australia), Dr Claudine van De Venter (University of Capetown) and Ms Randa Karzon (Endeavour Collage of Natural Therapies). We are pleased to share a common goal as researchers in being committed to strengthening the development of meaningful and practice relevant research in complementary healthcare.

 **Inclusion/exclusion criteria**
 Before you decide to participate in this research study, we need to ensure that it is ok for you to take part. You are eligible to participate in this study if you are: A qualified naturopath, Western herbalist, or nutritionist (not Accredited Practicing Dietitian) currently in clinical practice in Australia. A current member of a professional association relevant to your clinical qualification (i.e., naturopathy, Western herbal medicine, or nutrition [not Accredited Practicing Dietitian]). A provider of healthcare to individuals with mental health problems. 
 **Do I have to take part in this research study?**
Participation in this study is voluntary. It is completely up to you whether or not you decide to take part. If you decide to participate, we will invite you to complete a survey online that will take approximately 15 minutes to complete. You will be asked a series of questions that relate to the clinical care you provide to patients with mental health problems, and the characteristics of your clinical practice and education.

 You can change your mind at any time and stop completing the survey without consequences.

 By completing the survey, it is assumed you consent to take part and are telling us that you: Understand what you have read, Agree to take part in the research study as outlined below, Agree to the use of the survey data as described.
 **Are there any risks/inconvenience?**
 We don’t expect this questionnaire to cause any harm or discomfort, however if you experience feelings of distress as a result of participation in this study you can let the researcher know and they will provide you with assistance.

 **What will happen to information about me?**
 Access to the online questionnaire is via the red button at the end of this information statement. Submission of the online questionnaire is an indication of your consent. By clicking the submit button you consent to the research team collecting and using personal information about you for the research project. All this information will be treated confidentially. As this study will not collect identifying information you cannot be identified from your survey responses. The information collected will be stored securely within an online survey platform and electronic data files saved in secure (password protected) cloud-based storage that only the lead researcher (Dr Erica McIntyre) has access to. All data will be retained for five years after the study. Your information will only be used for the purpose of this research project.

 We plan to publish the results in peer-reviewed academic publications and present at complementary medicine conferences to clinicians, educators and researchers as we anticipate the findings will help inform undergraduate and postgraduate education and the clinical care provided to people with mental health problems. As the data will be de-identified when collected, it is not possible for the research team to report results directly back to participants; however, findings from this project will be shared with media outlets for publication in the general media.

 **What if I have concerns or a complaint?**
 If you have concerns about the research that you think I can help you with, please feel free to contact me on email at erica.mcintyre@uts.edu.au or phone at 02 9514 5942.  

 If you would like to talk to someone who is not connected with the research, you may contact the Research Ethics Officer on 02 9514 9772 or Research.ethics@uts.edu.au and quote this number **ETH19-3477**.

End of Block: Informed Consent

Start of Block: Screening questions

QS1 Are you a practicing naturopath, Western herbalist or nutritionist (not Accredited Practicing Dietitian)?

- Yes (1)
- No (2)

Skip To: End of Survey If QS1 = 2

QS2 Are you a member of a complementary medicine professional association?

- Yes (1)
- No (2)

Skip To: End of Survey If QS2 = 2

QS3 Do you provide health care to patients for their mental health?

- Always (1)
- Very Often (2)
- Sometimes (3)
- Rarely (4)
- Never (5)

Skip To: End of Survey If QS3 = 5

End of Block: Screening questions

Start of Block: Definition of terms used in this study

DEF **Definition of terms used in this study**   
In this study “**mental disorders**” are defined as per the Diagnostic and Statistical Manual for Mental Disorders version 5 (DSM-5). “**Mental health problems**” are defined as clinically meaningful mental health symptoms experienced by people that have not been diagnosed with a mental disorder, which includes subclinical depression or anxiety.

End of Block: Definition of terms used in this study

Start of Block: Care seeking for mental health

S1 **Care seeking for mental health** In this section, we would like to know what types of mental health problems or mental disorders people are consulting you for.

QS1.1 *Select the most appropriate response(s) to the following:* People **seek my care** specifically to treat the following **mental health problems** (i.e., not diagnosed disorders).
 *Select all that apply.* “**Mental health problems**” are defined as clinically meaningful mental health symptoms experienced by people that have not been diagnosed with a mental disorder, which includes subclinical depression or anxiety.

|  | Always (1) | Very often (2) | Sometimes (3) | Rarely (4) | Never (5) |
| --- | --- | --- | --- | --- | --- |
| Anxiety symptoms (1) |  |  |  |  |  |
| Depression symptoms (2) |  |  |  |  |  |
| Psychological stress/distress (3) |  |  |  |  |  |
| Sleep problems (4) |  |  |  |  |  |
| To improve or maintain general mental wellbeing (5) |  |  |  |  |  |
| Other mental health problems (not diagnosed disorders) not listed (6) |  |  |  |  |  |

| Page Break |  |
| --- | --- |

QS1.2 People **seek my care** specifically to treat the following diagnosed **mental disorders.** 
*Select all that apply.*
 
“**Mental disorders**” are defined as per the *Diagnostic and Statistical Manual for Mental Disorders version 5*(DSM-5).

- People do not seek my care specifically to treat diagnosed mental disorders (32)
- Acute stress disorder (1)
- Alcohol use disorder (29)
- Attention-deficit hyperactivity disorder (17)
- Autism spectrum disorder (16)
- Bipolar I (10)
- Bipolar II (11)
- Conduct disorder (20)
- Encopresis (26)
- Enuresis (25)
- Feeding and eating disorders (e.g. anorexia nervosa and bulimia nervosa) (22)
- Generalised anxiety disorder (4)
- Hypersomnolence disorder (14)
- Insomnia disorder (13)
- Major depressive disorder (7)
- Obsessive-compulsive disorder (23)
- Oppositional defiant disorder (19)
- Panic disorder (5)
- Persistent depressive disorder (dysthymia) (8)
- Post-traumatic stress disorder (2)
- Schizophrenia spectrum and other psychotic disorders (28)
- Other anxiety disorder (not listed) (6)
- Other bipolar related disorders (not listed) (12)
- Other depressive disorders (not listed) (9)
- Other disruptive, impulse-control and conduct disorders (not listed) (21)
- Other elimination disorders (not listed) (27)
- Other neurodevelopmental disorders (not listed) (18)
- Other obsessive-compulsive related disorders (not listed) (24)
- Other sleep-wake disorders (not listed) (15)
- Other substance-related and addictive disorders (not listed) (30)
- Other trauma and stress-related disorders (3)
- Other diagnosed mental disorders (please specify) (31) ________________________________________________

| Page Break |  |
| --- | --- |

QS1.3 What do you believe the expectations of your patients are when they first come to see you for mental health problems/disorders?
 *Select all that apply.*

- To “cure” or “fix” them (1)
- To complement standard care provided by their doctor or specialist (2)
- To reduce the number of prescription medications they are taking (3)
- To replace the prescription medications they are taking (4)
- To reduce the risk of developing more serious mental illness (5)
- To better understand their mental health (6)
- To receive an alternative opinion about their treatment options (7)
- Other (please specify) (8) ________________________________________________

| Page Break |  |
| --- | --- |

QS1.4 On average, how many patients do you provide care for **per week** for mental health **problems** (not diagnosed disorders)?

- 0 (1)
- 1–3 (2)
- 4–6 (3)
- 7–10 (4)
- 11 or more (5)

QS1.5 On average, how many patients do you provide care for **per week** for medically diagnosed mental **disorders**?

- 0 (1)
- 1–3 (2)
- 4–6 (3)
- 7–10 (4)
- 11 or more (5)

End of Block: Care seeking for mental health

Start of Block: Section 2: Clinical management of mental health

S2.A **Clinical management of mental health**
This section is focused on the management of mental health in your clinical practice, in particular, the most common mental health problems/disorders of depression, anxiety and stress.

QS2.1 What **clinical techniques** do you use to assess and monitor your patient’s mental health?
 *Select all that apply*

- Bioelectrical Impedance Analysis (1)
- Case history/notes based on patient self-report (2)
- Pathology testing (functional or conventional blood markers) (3)
- Iridology (4)
- Live blood analysis (5)
- Symptom-based questionnaires provided by product suppliers (6)
- Validated psychometric scales (e.g., Kessler Psychological Distress Scale [K10], Depression Anxiety Stress Scale [DASS]) (7)
- Vega testing (8)
- Other (please specify) (9) ________________________________________________

| Page Break |  |
| --- | --- |

Display This Question:

If QS2.1 = 3

QS2.2 What**pathology tests** have you used to assess and monitor your patient’s mental health? 
*Select all that apply*

- Active B12 (1)
- C-reactive protein (CRP) (2)
- Faecal microbial testing (3)
- Fasting homocysteine (4)
- Full blood examination (5)
- Gene profile (including MTHFR) (6)
- Hair analysis (heavy metals) (7)
- IgG4 food intolerance testing (8)
- Iron studies (9)
- Liver function tests (10)
- Plasma or serum zinc (11)
- Salivary Hormone Profiles (12)
- Salivary Neurotransmitter Profiles (13)
- Plasma or serum copper (14)
- Serum or RBC folate (15)
- Thyroid function tests and/or thyroid auto-antibodies (16)
- Urea and electrolytes (17)
- Urinary organic acid profiles (18)
- Vitamin D (19)
- Other (please specify) (20) ________________________________________________

| Page Break |  |
| --- | --- |

Display This Question:

If QS2.1 = 6

QS2.3 What **symptom-based questionnaires** (provided by product suppliers) have you used to assess and monitor your patient’s mental health?
*Please describe here.*

________________________________________________________________

________________________________________________________________

________________________________________________________________

________________________________________________________________

________________________________________________________________

Display This Question:

If QS2.1 = 7

QS2.4 Which of the following **validated psychometric scales** have you used to assess and monitor your patient’s mental health?
 *Select all that apply*

- Australian Quality of Life Instrument (AQoL-4D, AQoL-6D or AQoL-8D) (1)
- Depression Anxiety and Stress Scale (DASS) (2)
- Click to write Choice 11 (11)
- Geriatric Depression Scale (GDS) (3)
- Hamilton Anxiety Rating Scale (HAM-A) (4)
- Hamilton Depression Rating Scale (HAM-D) (5)
- Kessler Psychological Distress Scale (K10) (6)
- Patient Health Questionnaire (PHQ-2, PHQ-9, GAD-7) (7)
- Perceived Stress Scale (PSS) (8)
- Short Form Health Survey (SF-12, SF-20, SF-36) (9)
- Other (please specify) (10) ________________________________________________

End of Block: Section 2: Clinical management of mental health

Start of Block: Section 2b: Clinical management of mental health: treatments used

S2.B Clinical management of mental health: treatments used
This section is focused on the management of mental health in your clinical practice, in particular, the treatments you use/prescribe for the mental health problems/disorders of depression, anxiety and stress.

QS2.5 For each **herbal medicine** listed please **select each condition** you prescribe that herb for (i.e., anxiety, depression and/or stress). 
 *Select all that apply*.

|  | Anxiety (1) | Depression (2) | Stress (3) | I do not prescribe this herb for anxiety, stress or depression (4) |
| --- | --- | --- | --- | --- |
| Bacopa monnieri (brahmi) (1) |  |  |  |  |
| Centella asiatica (gotu kola) (2) |  |  |  |  |
| Crocus sativa (saffron) (3) |  |  |  |  |
| Curcuma longa (turmeric) (4) |  |  |  |  |
| Eleutherococcus senticosus (Siberian ginseng) (5) |  |  |  |  |
| Eschscholzia californica (California poppy) (6) |  |  |  |  |
| Ginkgo biloba (ginkgo) (7) |  |  |  |  |
| Glycyrrhiza glabra (licorice) (8) |  |  |  |  |
| Humulus lupulus (hops) (9) |  |  |  |  |
| Hypericum perforatum (St. John’s wort) (10) |  |  |  |  |
| Lavendula angustifolia (lavender) (11) |  |  |  |  |
| Matricaria recutita (chamomile) (12) |  |  |  |  |
| Melissa officinalis (lemon balm) (13) |  |  |  |  |
| Panax ginseng (Korean ginseng) (14) |  |  |  |  |
| Passiflora incarnata (passionflower) (15) |  |  |  |  |
| Piper methysticum (kava) (16) |  |  |  |  |
| Rehmania glutenosa (Rehmania) (17) |  |  |  |  |
| Rhodiola rosea (Rhodiola) (18) |  |  |  |  |
| Rosmarinus officinalis (rosemary) (19) |  |  |  |  |
| Scutellaria lateriflora (skullcap) (20) |  |  |  |  |
| Valeriana officinalis (valerian) (21) |  |  |  |  |
| Withania somnifera (ashwagandha) (22) |  |  |  |  |
| Ziziphus spinosa (ziziphus) (23) |  |  |  |  |
| Other (please specify) (26) |  |  |  |  |

| Page Break |  |
| --- | --- |

QS2.6 For each **nutritional supplement** listed please **select each condition** you prescribe that supplement for (i.e., anxiety, depression and/or stress). 
 *Select all that apply.*

|  | Anxiety (1) | Depression (2) | Stress (3) | I do not prescribe this supplement for anxiety, stress or depression (4) |
| --- | --- | --- | --- | --- |
| 5-hydroxytryptophan (5-HTP) (1) |  |  |  |  |
| B complex vitamins (2) |  |  |  |  |
| Calcium (3) |  |  |  |  |
| Extemporaneous compounding (16) |  |  |  |  |
| Folic acid (4) |  |  |  |  |
| Glutamine (5) |  |  |  |  |
| Magnesium (6) |  |  |  |  |
| Omega 3 fatty acids (e.g. fish oil) (7) |  |  |  |  |
| S-adenosyl-L-methionine (SAMe) (8) |  |  |  |  |
| Theanine (9) |  |  |  |  |
| Vitamin B6 (10) |  |  |  |  |
| Vitamin B12 (11) |  |  |  |  |
| Zinc (12) |  |  |  |  |
| Other (please specify) (15) |  |  |  |  |

QS2.7 For each **complementary therapy** listed please **select each condition** you prescribe that therapy for (i.e., anxiety, depression and/or stress). 
 *Select all that apply.*

|  | Anxiety (1) | Depression (2) | Stress (3) | I do not use this therapy for anxiety, stress or depression (4) | I refer to another health practitioner if this therapy is recommended for anxiety, stress or depression (5) |
| --- | --- | --- | --- | --- | --- |
| Acupuncture (1) |  |  |  |  |  |
| Emotional freedom technique (2) |  |  |  |  |  |
| Flower essences (3) |  |  |  |  |  |
| Homeopathy (4) |  |  |  |  |  |
| Massage (5) |  |  |  |  |  |
| Myotherapy (6) |  |  |  |  |  |
| Meditation (other than mindfulness) (7) |  |  |  |  |  |
| Mindfulness-based techniques (8) |  |  |  |  |  |
| Reiki (9) |  |  |  |  |  |
| Tai chi (10) |  |  |  |  |  |
| Yoga (11) |  |  |  |  |  |
| Other (please specify) (14) |  |  |  |  |  |

| Page Break |  |
| --- | --- |

QS2.8 For each **psychological therapy** listed please s**elect each condition** you prescribe that therapy for (i.e., anxiety, depression and/or stress). 
 *Select all that apply.*

|  | Anxiety (1) | Depression (2) | Stress (3) | I do not use this therapy for anxiety, stress or depression (4) | I refer to another health practitioner if this therapy is recommended for anxiety, stress or depression (5) |
| --- | --- | --- | --- | --- | --- |
| Behavioural activation (1) |  |  |  |  |  |
| Cognitive behavioural therapy (2) |  |  |  |  |  |
| Environmental health counselling (3) |  |  |  |  |  |
| Goal setting (4) |  |  |  |  |  |
| Motivational interviewing (5) |  |  |  |  |  |
| Physical activity counselling (6) |  |  |  |  |  |
| Psychoeducation (7) |  |  |  |  |  |
| Psychological counselling (psychotherapy) (8) |  |  |  |  |  |
| Sleep hygiene counselling (9) |  |  |  |  |  |
| Other (please specify) (12) |  |  |  |  |  |

| Page Break |  |
| --- | --- |

End of Block: Section 2b: Clinical management of mental health: treatments used

Start of Block: Section 2c: Clinical management of mental health: treatment decision-making

S2.C Clinical management of mental health: treatment decision-making
This section is focused on the management of mental health in your clinical practice, in particular, how you manage your treatment decision-making for the mental health problems/disorders of depression, anxiety and stress.

QS2.9 **How often** do you treat patients who are currently taking **prescription medications** specifically for a mental disorder or mental health problems?

- Always (1)
- Very Often (2)
- Sometimes (3)
- Rarely (4)
- Never (5)

| Page Break |  |
| --- | --- |

QS2.10 When treating anxiety, depression or stress, **how often** do you consult a drug-herb or drug-nutrient interaction resource (e.g. book or database) to ascertain the safety of any treatment combinations?

- Always (1)
- Very Often (2)
- Sometimes (3)
- Rarely (4)
- Never (5)

| Page Break |  |
| --- | --- |

QS2.11 What **resources** do you use to determine potential drug-herb or drug-nutrient interactions when treating anxiety, depression or stress?
 *Select all that apply*

- IM Gateway database (1)
- Medline plus database (2)
- Natural Medicines database (3)
- Other database (please specify) (11)
- General web search (7)
- Journal articles (5)
- Product manufacturer website (6)
- Talk to pharmacist (8)
- Text book (9)
- Other resource (please specify) (12) ________________________________________________

| Page Break |  |
| --- | --- |

QS2.12 Have you ever had to seek emergency assistance for a patient with mental health problems?

- Yes (1)
- No (2)

Display This Question:

If QS2.12 = 1

QS2.13 What emergency assistance did you seek? 
 *Select all that apply*

- Emergency services (000) (1)
- Lifeline (13 11 14) (2)
- Treating general practitioner (3)
- Treating psychologist (4)
- Treating psychiatrist (5)
- Other (please specify) (6) ________________________________________________

Display This Question:

If QS2.12 = 1

QS2.14 How did you determine that the patient needed emergency assistance?

________________________________________________________________

________________________________________________________________

________________________________________________________________

________________________________________________________________

________________________________________________________________

End of Block: Section 2c: Clinical management of mental health: treatment decision-making

Start of Block: Section 3: Interprofessional communication

S3 **Interprofessional communication** In this section, we would like to know your opinion about the way complementary medicine practitioners (i.e., naturopaths, Western herbalists and nutritionists) communicate and share medical information with other health care professionals.

QS3.1 *Communication between complementary medicine practitioners and* ***doctors****.*

 Please indicate your level of agreement with the following statements.

|  | Disagree (1) | Somewhat disagree (2) | Somewhat agree (3) | Agree (4) |
| --- | --- | --- | --- | --- |
| Doctors and complementary medicine practitioners share medical information received from or delivered to the patient (1) |  |  |  |  |
| Doctors inform complementary medicine practitioners of medical information that was delivered to patients (2) |  |  |  |  |
| Doctors and complementary medicine practitioners collaborate to decide what medical information should be delivered to patients (3) |  |  |  |  |
| It is easy for me to discuss patients with doctors (4) |  |  |  |  |
| There are barriers to communicating with doctors (5) |  |  |  |  |

| Page Break |  |
| --- | --- |

QS3.2 *Communication between complementary medicine practitioners and* ***specialist mental health practitioners*** *(i.e. psychologists or psychiatrists).*

 Please indicate your level of agreement with the following statements.

|  | Disagree (1) | Somewhat disagree (2) | Somewhat agree (3) | Agree (4) |
| --- | --- | --- | --- | --- |
| Specialist mental health care practitioners and complementary medicine practitioners share medical information received from or delivered to the patient (1) |  |  |  |  |
| Specialist mental health care practitioners inform complementary medicine practitioners of medical information that was delivered to patients (2) |  |  |  |  |
| Specialist mental health care practitioners and complementary medicine practitioners collaborate to decide what medical information should be delivered to patients (3) |  |  |  |  |
| It is easy for me to discuss patients with specialist mental health care practitioners (4) |  |  |  |  |
| There are barriers to communicating with specialist mental health care practitioners (5) |  |  |  |  |

| Page Break |  |
| --- | --- |

QS3.3 When patients are under your care for anxiety, depression or stress, **how often** do you refer them to the following health practitioners?

|  | Always (1) | Often (2) | Sometimes (3) | Seldom (4) | Never (5) |
| --- | --- | --- | --- | --- | --- |
| Doctor (1) |  |  |  |  |  |
| Psychologist (2) |  |  |  |  |  |
| Psychiatrist (3) |  |  |  |  |  |
| Counsellor (4) |  |  |  |  |  |
| Other (please specify) (5) |  |  |  |  |  |

Skip To: End of Block If QS3.3 = 5

| Page Break |  |
| --- | --- |

QS3.4 How do you initiate the referral of a patient with anxiety, depression or stress to other health practitioners?
 *Select all that apply.*

- Letter to health practitioner sent with patient (1)
- Letter sent directly to health practitioner (4)
- Other (please specify) (5) ________________________________________________

End of Block: Section 3: Interprofessional communication

Start of Block: Section 4a: Qualifications and training in mental health

S4a **Qualifications and training in mental health**
 In this section we would like to know what training you have undertaken in mental health.

QS4.1 What **formal qualifications** do you have in mental health?
 A formal qualification is defined as a qualification (diploma or above) you have obtained in an area of clinical mental health.
 *Select all that apply.*

- Diploma in Counselling (1)
- Advanced Diploma in Counselling (2)
- 3-year Bachelor Degree in Counselling (3)
- 3-year Bachelor Degree in Psychology (4)
- 4-year Bachelor Degree in Psychology (5)
- Other Bachelor degree related to mental health (please specify) (6) ________________________________________________
- Graduate Certificate in mental health / Counselling (7)
- Graduate Diploma in mental health / Counselling (8)
- Masters in Psychology (Health, Clinical, Neuropsychology, or Counselling) (9)
- PhD in Psychology (10)
- Other (please specify) (11) ________________________________________________
- I have no formal qualifications in mental health (12)

| Page Break |  |
| --- | --- |

QS4.2 What other mental health **training** have you completed (e.g. workshops, seminars, short courses)?
*Select all that apply.*

- Mental Health First Aid (1)
- Industry led seminar/workshop (2)
- Association led seminar/workshop (3)
- Academic conference workshop (4)
- Association led conference workshop (5)
- Other (please specify) (6) ________________________________________________
- I have no other mental health training (7)

| Page Break |  |
| --- | --- |

Q62 Have you had **formal training** in any of the following psychological therapies?
Formal training is delivered as a "course" of work that results in a qualification (certificate or above) *or* as part of a clinical qualification (e.g. Masters of Clinical Psychology).
*Select all that apply.*

- Behavioural activation (6)
- Cognitive behavioural therapy (4)
- Goal setting (5)
- Mindfulness-based techniques (1)
- Motivational interviewing (10)
- Other (please specify) (8)
- I have no other formal training in psychological therapies (9)

| Page Break |  |
| --- | --- |

QS4.3 What resources do you use to assist your clinical decision-making when managing patients with mental health problems/disorders?
 *Select all that apply.*

- Clinical guidelines (e.g., RACGP, NICE) (4)
- Diagnostic and Statistical Manual for Mental Disorders (DSM) (2)
- Industry provided technical information (6)
- International Classification of Diseases (ICD) (3)
- Peer-reviewed journal articles (1)
- Text books (5)
- Other (please specify) (7) ________________________________________________

End of Block: Section 4a: Qualifications and training in mental health

Start of Block: Block 9

QS4.4 **Qualifications and training in mental health.**
In this section, we would like to know more about how well you believe your education has prepared you for treating mental health problems/disorders.   Reflecting on your time in clinical practice, please indicate **your level of agreement** with the following statements on a scale of *1 = Strongly disagree to 5 = Strongly agree.*

|  | Strongly disagree (1) | Somewhat disagree (2) | Neither agree nor disagree (3) | Somewhat agree (4) | Strongly agree (5) |
| --- | --- | --- | --- | --- | --- |
| My undergraduate qualification contained sufficient content on mental health (1) |  |  |  |  |  |
| My undergraduate qualification adequately prepared me to manage mental health problems/disorders in clinical practice (2) |  |  |  |  |  |
| I would have preferred to have undertaken more training in mental health in my undergraduate education (3) |  |  |  |  |  |
| I would like to undertake postgraduate training in mental health (4) |  |  |  |  |  |

| Page Break |  |
| --- | --- |

QS4.5 How confident are you in your knowledge and skills to treat/manage the following mental health problems/disorders?
 Please rate your **level of confidence** on a scale from 1 = *Not at all confident* to 5 = *highly confident*.

|  | Not at all confident (1) | 2 (2) | 3 (3) | 4 (4) | Highly confident (5) |
| --- | --- | --- | --- | --- | --- |
| Anxiety symptoms (1) |  |  |  |  |  |
| Anxiety disorders (2) |  |  |  |  |  |
| Bipolar related disorders (3) |  |  |  |  |  |
| Depression symptoms (4) |  |  |  |  |  |
| Depressive disorders (5) |  |  |  |  |  |
| Eating disorders (6) |  |  |  |  |  |
| General mental wellbeing (7) |  |  |  |  |  |
| Insomnia disorder (8) |  |  |  |  |  |
| Psychotic disorders (9) |  |  |  |  |  |
| Sleep problems (10) |  |  |  |  |  |
| Psychological stress-related symptoms (11) |  |  |  |  |  |

| Page Break |  |
| --- | --- |

QS4.6 Please rate your **level of confidence** on a scale from 1 = *Not at all confident* to 5 = *highly confident*.

|  | Not at all confident (1) | 2 (2) | 3 (3) | 4 (4) | Highly confident (5) |
| --- | --- | --- | --- | --- | --- |
| How confident are you in your ability to assess a patient for suicide risk? (1) |  |  |  |  |  |

| Page Break |  |
| --- | --- |

QS4.7
What do you believe to be the **amount of suicide risk** for the following diagnoses?
 Please indicate the option that best applies to you for each condition on a scale from 1 = *low risk* to 5 = *high risk*.

|  | Low risk (1) | 2 (2) | 3 (3) | 4 (4) | High risk (5) |
| --- | --- | --- | --- | --- | --- |
| Post-traumatic stress disorder (1) |  |  |  |  |  |
| Bipolar disorder (2) |  |  |  |  |  |
| Major depression (3) |  |  |  |  |  |
| Substance abuse (4) |  |  |  |  |  |
| Generalised anxiety disorder (5) |  |  |  |  |  |
| Impulse control disorders (6) |  |  |  |  |  |
| Psychotic disorders (7) |  |  |  |  |  |

| Page Break |  |
| --- | --- |

End of Block: Block 9

Start of Block: Section 5: Demographics and practice characteristics

QS5.1 What state or territory is your **primary** practice located?

- Australian Capital Teritory (1)
- New South Wales (2)
- Northern Territory (3)
- Queensland (5)
- South Australia (4)
- Tasmania (6)
- Victoria (7)
- Western Australia (8)

| 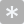 |
| --- |

QS5.2 What is the postcode of your **primary** practice location?

________________________________________________________________

| Page Break |  |
| --- | --- |

| 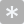 |
| --- |

QS5.3 What is your year of birth?

________________________________________________________________

| Page Break |  |
| --- | --- |

QS5.4 What is your gender?

- Male (1)
- Female (2)
- Other (3)

| Page Break |  |
| --- | --- |

QS5.5 What is your **primary** clinical discipline?

- Naturopathy (1)
- Western herbal medicine (2)
- Nutrition (not Accredited Practicing Dietitian) (3)
- Other (please specify) (4) ________________________________________________

| Page Break |  |
| --- | --- |

QS5.6 Please select **all** degrees/qualifications you hold **relevant to your clinical practice**.

- Certificate IV (1)
- Diploma (2)
- Advanced diploma (3)
- Bachelor's degree (4)
- Graduate certificate (5)
- Graduate diploma (6)
- Masters degree (7)
- Professional Doctorate (e.g., Doctor of Naturopathy/ND) (8)
- Doctor of Philosophy (PhD) (9)

| Page Break |  |
| --- | --- |

QS5.7 How many **years** ago did you receive your **highest** complementary medicine qualification (i.e., naturopathy, Western herbalism or nutrition)?

- Less than 5 (1)
- 5-9 (2)
- 10-14 (3)
- 15-19 (4)
- 20 or more (5)

| Page Break |  |
| --- | --- |

QS5.8 What other clinical qualifications do you hold?
 *Select all that apply*

- I do not have any other clinical qualifications (5)
- Nursing (1)
- Medical (2)
- Other allied health (please specify) (3) ________________________________________________
- Other complementary medicine (please specify) (4) ________________________________________________

Skip To: QS5.9 If QS5.8 = 5

| Page Break |  |
| --- | --- |

QS5.9 What educational institution/s did you obtain your **undergraduate** complementary medicine (i.e., naturopathy, Western herbalism or nutrition) clinical qualification/s from?
 *Select all that apply*

- Australasian College of Natural Therapies (1)
- Charles Sturt University (2)
- Endeavour College of Natural Health (3)
- Nature Care College (4)
- Paramount College (5)
- Southern School of Natural Therapies (6)
- Southern Cross University (7)
- University of New England (8)
- WEA Hunter (9)
- Western Sydney University (10)
- Other University/College (please specify) (11) ________________________________________________
- Other overseas institution (please specify) (12) ________________________________________________

| Page Break |  |
| --- | --- |

QS5.10 What type of clinical setting/s to you practice in?
 *Select all that apply.*

- Solo practitioner (1)
- Practice with other complementary medicine providers (2)
- Practice with other conventional health care providers (3)
- Integrative practice with a mix of complementary medicine and conventional health care providers (4)
- Within an educational setting (i.e. student clinic) (5)
- Other (please specify) (6) ________________________________________________

| Page Break |  |
| --- | --- |

QS5.11 Please provide any additional comments about the management of mental health problems/disorders in complementary medicine practice, or about the survey itself.

________________________________________________________________

End of Block: Section 5: Demographics and practice characteristics

Start of Block: End

Q61
**Thank you for taking the time to complete this survey.**

 Please click the link below if you would like to enter the prize draw to win one of 14 gift vouchers valued at $50. 
Your email address will not be linked to your survey responses which will remain anonymous.
 <https://utshealth.qualtrics.com/jfe/form/SV_0NVSMFCdgesW7gF>

End of Block: End
